# Factors That Influence Actual Playing Time: Evidence From the Chinese Super League and English Premier League

**DOI:** 10.3389/fpsyg.2022.907336

**Published:** 2022-07-04

**Authors:** Yuangang Zhao, Tianbiao Liu

**Affiliations:** ^1^School of Sports and Health, Huaihua University, Huaihua, China; ^2^College of Physical Education and Sports, Beijing Normal University, Beijing, China

**Keywords:** playing time, Chinese Super League, English Premier League, football (soccer), soccer (football), game stoppage time

## Abstract

This study explored factors that influence actual playing time by comparing the Chinese Super League (CSL) and English Premier League (EPL). Eighteen factors were classified into anthropogenic and non-anthropogenic factors. Fifty CSL matches (season 2019) and 50 EPL matches (season 2019–2020) were analyzed. An independent sample t-test with effect size (Cohen’s d) at a 95% confidence interval was used to evaluate differences in the influencing factors between the CSL and EPL. Two multiple linear regression models regarding the CSL and EPL were conducted to compare the influencing factors’ impact on actual playing time. The results showed that the average actual playing time (*p* < 0.05, 0.6 < ES = 0.610 < 1.2) and average game density (*p* < 0.05, 0.2 < ES = 0.513 < 0.6) in the EPL were significantly higher than in the CSL. The average time per game for general fouls (*p* < 0.05, 1.2 < ES = 1.245 < 2.0) and minor injuries (*p* < 0.05, 0.2 < ES = 0.272 < 0.6) in the CSL was significantly higher than in the EPL. The average time allocated to off-field interferences in the CSL was significantly higher than in the EPL, while the average time allocated to throw-ins (out-of-bounds) in the CSL was significantly lower than in the EPL (*p* < 0.05, 0.2 < ES = 0.556 < 0.6). The study showed that actual playing time in CSL games was more affected by anthropogenic factors than in the case of EPL games, while both leagues were equally affected by non-anthropogenic factors. This study provides a reference for coaches to design effective training and formulate game strategies for elite soccer leagues.

## Introduction

In a soccer game, actual playing time is defined as the duration of play after subtracting the time taken up by stoppages, substitutions, goals, injuries, and other incidences (Castellano et al., 2011), which are added on as extra time at the end of the game. The Fédération Internationale de Football Association (FIFA) calculates the actual playing time to assess the continuity, quality, and watchability of a game (Peng, 2017) as it is an important indicator of the level of a league. The actual playing time of a soccer game can be considerably less than 90 min due to stoppages caused by free kicks, corner kicks, substitutions, goals, player injuries, and other interferences (Cook and Goff, 2006; Lago-Peñas et al., 2012). The level of the players, the match status of the game, the level of play, and the team’s playing style (Boyko et al., 2007; Dong, 2012; Greve et al., 2019) can also have an impact. All these factors can increase the total playing time. The use of a video assistant referee (VAR) in recent years has led to more stoppages, resulting in an increase in total playing time (Han et al., 2020).

An elite soccer games can be stopped approximately 108 times and account for up to 38% of the total game (Siegle and Lames, 2012a). Stoppages during a game have a significant impact on a player’s performance. Previous studies have found that stoppages cause extended extra time and reduce players’ running performance (Linke et al., 2018); moreover, running distance in the second half of a game may decrease because of too many stoppages rather than a decrease in physical capacity (Carling and Dupont, 2011; Rey et al., 2020). Some soccer teams will deliberately create opportunities for stoppages (place kicks, out-of-bound goals, etc.) when they are ahead or stall at dead balls (Siegle and Lames, 2012a,b; Augste and Cordes, 2016) to disrupt the continuity of the other team’s attack, especially in the final stages of a game (Carling and Dupont, 2011).

Stoppages are directly associated with some of the indicators of soccer games and play an important guiding role in the time, frequency, density, and intervals of team training. Chinese soccer is gaining more public attention and social influence, and the Chinese Super League’s (CSL) games have increased in both pace and quality (China Football Federation, 2019). For example, the CSL’s average actual playing time in the 2008 season was 51 min and 25 s (Dong, 2012), and it rose to 54 min in the 2014–2017 seasons (Zhao, 2019). Nevertheless, the CSL’s actual playing time is still shorter than the world’s elite leagues. In 2006, the average actual playing time of the 18th FIFA World Cup was 60 min and 36 s, with an average match density of 62%. The 13th European Football Championship in 2008 had an average actual playing time of 62 min and 23 s. Since the CSL players have a lower load and match intensity than the five major European soccer leagues (Li, 2007), they cannot perform high-quality technical and tactical movements in high-intensity matches to produce a satisfactory performance.

The CSL is growing rapidly and the amount of research on the CSL is increasing (Jiang, 2013; He, 2016; Liu and Hopkins, 2017; Li, 2019), yet few studies have focused on the factors that impact actual playing time. The English Premier league (EPL)—a well-established league—is known as one of the best soccer leagues in the world; the CSL league—a developing league—has modeled itself on the EPL. Thus, this study aims to understand the nature of soccer from the two different contexts and compares the important factors that influence the actual playing time in the CSL and EPL, as the two typical leagues in the East and West. The results of this study may assist coaches in understanding the causes for stoppages in soccer games. Moreover, this study may offer a realistic guide for the coaches of professional clubs to design training programs that improve players’ ability under pressure and help referees to control different situations during a game.

## Materials and Methods

### Sample and Variables

This study randomly selected 50 matches in the CSL during the 2019 season and 50 matches in the EPL during the 2019–2020 season, prior to the 30th round. Matches after the 30th round were excluded because some rules were changed due to the COVID-19 pandemic. The matches were recorded onto a hard drive and included all 16 teams in the CSL and all 20 teams in the EPL. Three matches from each league were selected for pre-testing, and expert interviews were conducted to refine the statistical concepts and measurements. Following previous studies (Li, 2007; Yang et al., 2018; Gai et al., 2019; Zhao, 2019; Han et al., 2020), 18 stoppage-related variables were determined, which were then categorized as either non-anthropogenic or anthropogenic factors (Table 1). Non-anthropogenic stoppage factors were defined as stoppages that resulted from the “FIFA Laws of the Game” and the nature of soccer games, whereas anthropogenic stoppage factors were defined as stoppages that resulted from the behavior of players and other people at the venue.

**Table 1:** Classification of factors of stoppages.

No	Category	Factors of stoppages	Definition
1	Non-anthropogenic factors	Out-of-bounds (X_1_)	The time consumed for out-of-bounds is calculated from the time the ball is outside of the playing field till the player of the opposing team kicks off the ball
2		Goal kick (X_2_)	The time consumed for the goal kick is calculated from the time the whole of the ball kicked by the attacking team player passes over the goal line till the player of the defending team kicks off the ball at the penalty area
3		Resumption of play after a goal (X_3_)	The time consumed for resumption of play after a goal is calculated from the time the ball enters the goal line till the player of the opposing team kicks off the ball at the centre spot
4		Corner kick (X_4_)	The time consumed for corner kick is calculated from the time the player of the defending team kicks off the ball out of his own goal line till the player of the opposing team kicks off the ball from the corner area
5		Substitution of player (X_5_)	The time consumed for substitution of player is calculated from the time there is a dead ball situation till a player is replaced and the play is restarted
6		General foul (X_6_)	The time consumed for a general foul is calculated by the time on fouls without disciplinary penalties and building a defensive wall
7		Offside (X_7_)	The time consumed for offside is calculated from the time the referee blows the whistle till the player of the opposing team kicks
8		Building a defensive wall (X_8_)	The time consumed for building a defensive wall is calculated from the time the referee blows the whistle of foul (the defending team building a defensive wall) till the ball is kicked off
9		VAR intervention (X_9_)	The time consumed for VAR intervention is calculated from the time the referee blows the whistle of foul (with VAR intervention) till the tall is kicked off by the opposing team player
10		Team doctor intervention (X_10_)	The time consumed for team doctor intervention is calculated from the time a player gets injured till the medical stoppage ends
11		Presentation of yellow card (X_11_)	The time consumed for presentation of yellow card is calculated from the time the referee blows the whistle and presents the yellow card till the play is restarted
12		Presentation of red card (X_12_)	The time consumed for presentation of red card is calculated from the time the referee blows the whistle and presents the red card till the play is restarted
13		Award of penalty kick (X_13_)	The time consumed for award of penalty kick is calculated from the time the referee blows the whistle and awards a penalty kick till the ball enters the goal
14		Dropped ball (X_14_)	The time consumed for dropped ball is calculated from the time the referee stops the play temporarily as necessary till he drops the ball to restart the play
15	Anthropogenic factors	Player minor injury (X_15_)	The time consumed for player minor injury is calculated from the time a player gets injured without the need of team doctor treatment till the play is restarted
16		Player conflict (X_16_)	The time consumed for player conflict is calculated from the time the referee stops the play due to a player conflict till the play is restarted
17		Off-field (X_17_)	The time consumed for off-field is calculated by the time on the stoppages caused by factors including the coaches, substitutes, officials, fans, etc.
18		Player complaint (X_18_)	The time consumed for player complaint is calculated by the time of the stoppage because a player may complain about the referee for not calling it a foul which a player reckons it is
19		Actual playing time	Actual playing time refers to the pure time of a soccer game. It includes 90 min plus stoppage time added and minus stoppage time. (actual playing time = 90 min + injury time–stoppage time)
20		Match density	Match density refers to the percentage of the actual playing time of the actual total time in a match.
21		Average total time consumption per match	Average total time consumption per match is the average time consumption derived from any of the 18 stoppage indicators

VAR, video assistant referee.

Afterwards, an experienced specialist examined the games and collected the data. The specialist recorded the time taken up by stoppages in the effective time of each game and exported the results onto Excel spreadsheets.

### Data Collection and Data Reliability

To test the reliability of the data, another experienced observer examined ten randomly selected games (10% of the sample: 5 CSL and 5 EPL games; Hernández-Moreno et al., 2011). Intraclass Correlation Coefficient (ICC) values ranged from 0.968 to 1.0 (Table 2), which exceeded 0.9 and thus proved excellent reliability (Koo and Li, 2016).

**Table 2:** Interclass correlation coefficient and correlation values for variables.

Independent variable		Intraclass correlation coefficient (ICC)
X_1_	Out-of-bounds	0.995
X_2_	Goal kick	0.990
X_3_	Resumption of play after a goal	0.968
X_4_	Corner kick	0.977
X_5_	Substitution of player	0.985
X_6_	General foul	0.995
X_7_	Offside	0.999
X_8_	Building a defensive wall	0.993
X_9_	VAR intervention	0.999
X_10_	Team doctor intervention	0.993
X_11_	Presentation of yellow card	0.996
X_12_	Presentation of red card	0.993
X_13_	Award of penalty kick	1.000
X_14_	Dropped ball	0.991
X_15_	Player minor injury	1.000
X_16_	Player conflict	0.999
X_17_	Off-field	×
X_18_	Player complaint	0.999

### Procedure and Statistical Analysis

A descriptive analysis of each variable was conducted for the two leagues. Data normality was calculated using the Kolmogorov–Smirnov test and was found to be normally distributed. Subsequently, an independent samples *t*-test (*p* < 0.05) was conducted to compare and analyze actual playing time, game density, duration of each game, and then tabulated 18 stoppage variables in the two leagues. The results were recorded as mean ± standard deviation (*M* ± SD). The effect size (ES, Cohen’s *d*) at 95% confidence intervals (CIs) was then used to measure the range of differences. ES was classified as follows: < 0.1 = insignificant difference, ≤ 0.2 = small difference, ≤ 0.6 = moderate difference, ≤ 1.2 = large difference, ≤ 2.0 = very large difference, and ≤ 4.0 = extremely large difference based on previous research (Hopkins et al., 2009; Zhou et al., 2019).

A multiple linear regression analysis was conducted to determine the differences in influencing factors between the CSL and EPL. In this analysis, the time consumed by each stoppage factor was taken as the independent variable, while the actual playing time was the dependent variable.


$$Y_{i}=a_{i1}x_{i1}+a_{i2}x_{i2}+...+b_{i}+\varepsilon_{i},$$

where i represents either the CSL or EPL, *b* is the constant term, and ε is the error term. The independent variables (*X*_1_–*X*_18_) and the dependent variable (*Y*) of the two leagues were subsequently inserted into the multiple linear regression model. In the results, the significance value, as the standardized coefficient, determined whether the independent variable (i.e., stoppage factors) contributed to the dependent variable (i.e., actual playing time). The beta value determined the degree of influence of the independent variables on the dependent variable. The analyses were done using IBM SPSS 22.0 (Statistical Package for the Social Sciences, SPSS Inc.). This study was complied according to the ethical principles stated by the Declaration of Helsinki.

## Results

### Comparison of Actual Playing Time and Stoppage Factors Between the CSL and EPL

Table 3 shows that the average actual playing time between the CSL and the EPL was statistically different (*p* = 0.03 < 0.05), and the effect size values showed a moderate difference (0.6 < ES = 0.610 < 1.2). The average match density (%) also showed a statistically significant difference (*p* = 0.008 < 0.05), the effect size values showed a minor difference (0.2 < ES = 0.513 < 0.6), and the average game time did not show any statistical difference.

**Table 3:** Comparison of average data of matches of the Chinese Super League (CSL) and English Premier League (EPL; time unit: minutes).

Factors/indicators			CSL (*n* = 50)		EPL (n = 50)		*T*-test on independent sample		
		*M*	SD	*M*	SD	*p*	ES	95% CI for Cohen’s d
	Actual playing time	52.91	5.35	56.24	5.19	0.003	0.610	± 0.207
	Match density (%)	54.76	5.88	57.70	5.58	0.008	0.513	± 0.113
	Playing time	96.68	1.52	97.24	2.08	0.151	0.165	± 0.061
Non-anthropogenic factors	Out-of-bounds (X1)	7.56	1.70	8.74	2.46	0.007	0.556	± 0.155
	Goal kick (X_2_)	6.64	2.10	5.96	1.93	0.092	0.193	± 0.400
	Resumption of play after a goal (X_3_)	3.23	1.77	3.43	1.73	0.746	0.038	± 0.187
	Corner kick (X_4_)	4.77	2.04	4.80	1.80	0.940	0.015	± 0.377
	Substitution of player (X_5_)	2.22	1.10	2.60	1.32	0.130	0.176	± 0.050
	General foul (X_6_)	7.85	2.53	5.11	2.13	<0.001	1.245	± 1.671
	Offside (X_7_)	0.82	0.88	1.05	0.77	0.063	0.215	±0.009
	Organization of wall (X_8_)	2.05	1.68	2.43	1.52	0.197	0.150	±0.076
	VAR intervention (X_9_)	1.43	1.42	1.11	1.21	0.324	0.113	±0.328
	Team doctor intervention (X_10_)	3.27	2.11	2.99	2.25	0.488	0.130	±0.523
	Presentation of yellow card (X_11_)	1.71	1.25	1.56	1.29	0.942	0.009	±0.232
	Presentation of red card (X_12_)	0.16	0.38	0.13	0.40	0.619	0.035	±0.257
	Award of penalty kick (X_13_)	0.48	0.92	0.36	0.64	0.699	0.037	±0.258
	Dropped ball (X_14_)	0.03	0.12	0.06	0.15	0.243	0.077	±0.663
Anthropogenic factors	Player minor injury (X_15_)	1.06	1.04	0.61	0.89	0.015	0.272	±0.468
	Player conflict (X_16_)	0.24	0.55	0.11	0.34	0.273	0.086	±0.303
	Off-field (X_17_)	0.04	0.13	0	0	0.042		
	Player complaint (X_18_)	0.17	0.49	0.06	0.24	0.126	0.283	±0.677

VAR, video assistant referee.

The average time spent on out-of-bounds between the CSL and the EPL was statistically different (*p* = 0.007 < 0.05), and the effect size showed a minor difference (0.2 < ES = 0.556 < 0.6). The average time spent on general fouls was also statistically different (*p* = 0.001 < 0.05), and the effect size showed a large difference (1.2 < ES = 1.245 < 2). The average time spent on players’ minor injuries was statistically different (*p* = 0.015 < 0.05), and the effect size showed a minor difference (0.2 < ES = 0.272 < 0.6). The time spent on field interferences by off-field factors was statistically different (*p* = 0.042 < 0.05).

### Comparison of Factors Impacting the Actual Playing Time Between the CSL and EPL

As is shown in Tables 4, 5, the models of actual playing time of CSL and EPL are significant. X_1_–X_11_ had an important impact on stoppage times for both the CSL and EPL. In the CSL, penalty kicks (X_13_), player conflict (X_16_), and player complaints (X_18_) influenced stoppage time, while in the EPL, minor player injuries were an impact factor. The remaining variables were not significant in the regression models.

**Table 4:** Linear regression coefficient for stoppage time and actual playing time.

Indicators	CSL			EPL		
	Beta	*t*	Sig.	Beta	*t*	Sig.
(Constant)		41.035	<0.001		52.961	<0.001
Out-of-bounds (X_1_)	−0.350	−7.535	<0.001	−0.493	−10.428	<0.001
Goal kick (X_2_)	−0.402	−7.883	<0.001	−0.347	−7.812	<0.001
Resumption of play after a goal (X_3_)	−0.317	−6.537	<0.001	−0.273	−5.690	<0.001
Corner kick (X_4_)	−0.397	−8.708	<0.001	−0.347	−7.818	<0.001
Substitution of player (X_5_)	−0.232	−4.807	<0.001	−0.246	−5.143	<0.001
General foul (X_6_)	−0.430	−8.888	<0.001	−0.389	−7.751	<0.001
Offside (X_7_)	−0.174	−3.775	0.001	−0.180	−3.711	<0.001
Organization of wall (X_8_)	−0.289	−6.998	<0.001	−0.311	−6.632	<0.001
VAR intervention (X_9_)	−0.161	−3.393	0.002	−0.118	−2.689	0.011
Team doctor intervention (X_10_)	−0.331	−7.526	<0.001	−0.203	−3.892	<0.001
Presentation of yellow card (X_11_)	−0.287	−5.169	<0.001	−0.099	−2.247	0.032
Presentation of red card (X_12_)	−0.066	−1.515	0.140	−0.057	−1.280	0.210
Award of penalty kick (X_13_)	−0.116	−2.370	0.024	−0.093	−1.721	0.095
Dropped ball (X_14_)	−0.020	−0.429	0.671	−0.062	−1.344	0.188
Player minor injury (X_15_)	−0.102	−1.948	0.061	−0.108	−2.336	0.026
Player conflict (X_16_)	−0.187	−3.735	0.001	−0.081	−1.875	0.070
Off-field (X_17_)	−0.021	−0.444	0.660			
Player complaint (X_18_)	−0.095	−2.095	0.044	−0.091	−1.851	0.073

Since the Off-field factor for the effect size (ES) shows zero, it is automatically not counted in the regression model.

VAR, video assistant referee.

**Table 5:** Model summary of actual playing time of CSL and EPL.

Model	*R*	*R* square	Adjusted *R* square	Std. Error of the estimate	Change statistics					Durbin-Watson
					*R* square change	*F* change	*df*1	*df*2	Sig. *F* change	
CSL	0.979	0.957	0.933	1.38761	0.957	38.792	18	31	<0.001	2.040
EPL	0.975	0.951	0.926	1.41616	0.951	36.844	17	32	<0.001	1.207

## Discussion

This study compared the factors that influenced actual playing time between the CSL and EPL. The results showed that the actual playing time in the CSL was less than in the EPL. The CSL spent more time on general fouls (X_6_), player minor injuries (X_15_), and off-field interferences (X_17_), while the EPL spent more time on out-of-bounds (X_1_). The factors that affected actual playing time also differed. For the CSL, anthropogenic factors dominated, and the primary factors included penalty kicks (X_13_), player conflict (X_16_), and player complaints (X_18_), while the primary factor for the EPL was minor player injuries (X_15_).

The CSL match density in the 2019 season reached 54.76%, slightly lower than for the EPL, which is generally consistent with the findings of studies by Augste and Lames (2008) and Siegle and Lames (2012a). Tschan et al. (2001) also found that in the European leagues, 32% of game time is consumed by stoppages; the same study found that in the World Cup, only 61–69% of the game time was dedicated to actual playing time. Similar to previous studies (Sun et al., 2014), in the CSL’s games, general fouls took more time and had a greater impact on actual playing time. Additionally, minor player injuries and off-field interruptions took more time. Out-of-bounds and throw-ins took more time in the EPL games than in CSL games. Another study found that the time consumed by free kicks and player injuries in CSL games in the 2008 season was higher than in the 13th European Football Championship in 2008 (Dong, 2012), which is consistent with the results of the current study. In other words, referees in the CSL stopped the game more frequently for minor fouls, which led to more interruptions to the game and a consequent increase in time consumption. Comparatively, the fluidity of the EPL games was better than in the CSL games, and referees were able to control the pace of the game and make good use of advantage rules. Moreover, a free kick after a foul in the CSL tended to be slightly slower due to various interferences, such as improper positioning or player blocking (Greve et al., 2019). Furthermore, players sometimes pretended to be injured after a foul, which increased the time consumed in a game.

Previous studies found that high-intensity games tend to be accompanied by longer intervals (Hernández-Moreno et al., 2011). Compared to other leagues, the EPL has a faster rhythm (Jamil and Kerruish, 2020); thus, players may take advantage of stoppages to recover from a high-intensity game, which can explain the greater time consumption during throw-ins in EPL games. In addition, Siegle and Lames (2012b) found that throw-ins in the defensive zone took more time. This may be because losing possession in the defensive zone could more easily lead to conceding a goal. Another possible reason may be that players face more intense running in the defensive zone and they want to recover from fatigue by taking opportunities to rest when the ball is out of play, like during throw-ins.

Stoppages in CSL games included off-field interferences, which were not found in the EPL games. Zhao and Zhang (2021) found that spectators also impacted the actual playing time by negatively affecting both players and referees. For example, referees in Major League Soccer (MLS) tend to add an extra 33 s when the home team is down by one goal (Yewell et al., 2014). EPL referees also demonstrate a preference for the home team (Boyko et al., 2007). In the CSL, home teams also enjoy an advantage (Peng et al., 2016; Liu et al., 2019) as the home teams are better off than the away team regarding offense indicators. Han et al. (2020) showed that after the introduction of objective VAR technology, home advantages conferred by referees decreased partially, based on the indicators for red and yellow cards and fouls, which were previously largely determined by referees’ vulnerability to the home team spectators. Although the use of new technology such as VAR has reduced home advantage in recent years (Han et al., 2020), the influence of spectators cannot be completely eliminated. Thus, a higher level of professional skills in both players and referees is required to reduce the impact of spectators.

In CSL games, penalty kicks, player conflict, and player complaints impacted actual playing time. Although there was no significant difference in total time compared to the EPL, these factors significantly impacted actual playing time in CSL games. Typically, when a penalty kick is awarded in CSL games, the player complains to the referee, which may result in the referee presenting a yellow card or even sending a player off the field. Players’ behaviors not directly related to the game actions, such as gathering and inter-player conflict, also waste time in a game. Previous studies have suggested that actual playing time in the CSL is likely influenced by referees’ ability to control the game, players’ professional and ethical qualities, the presentation of red and yellow cards, players disregarding referees’ decisions, players’ attitude toward the game, and players’ technical and tactical abilities (Jiang, 2013; Li and Lu, 2013; Sun et al., 2014; He, 2016; Li, 2019; Zhao, 2019). Penalty kicks, player conflict, and player complaints may reflect the players’ attitude to the game, or lack of technical and tactical ability; moreover, it has been suggested that players in the CSL are not afraid of being shown a second yellow card (Jamil and Kerruish, 2020). Such an attitude may lead to players committing fouls in the penalty area or being prone to taking excessive defensive actions that cause player conflict and penalties, or even lead to being sent off the field. Referees may also contribute to this situation. In CSL games, referees are often too hesitant to show a second yellow card (Mao et al., 2016), consequently, referees find it more difficult to act in time to control a situation on the field in the case of player conflict or player complaints, which encourages such behaviors.

The EPL games are typically played at a fast pace (Jamil and Kerruish, 2020), with fierce confrontation and frequent player minor injuries (Rahnama et al., 2002), especially in the first and last 15 min of the game. In the first 15 min, player injuries may result from intense player confrontation due to excitement, while player injuries in the last 15 min may be due to fatigue. Studies have also shown that stoppages are more likely to occur toward the end of a game (Carling and Dupont, 2011) and that many minor injuries are caused by fatigue. In contrast, more frequent stoppages and time consumption in the CSL dilute the intensity of a game; however, whether this reduces player injuries still needs to be examined in a further study.

Although this study provided significant findings, the study has a few limitations due to the small number of samples and the fact that contextual variables were not considered. Future studies should thus include these two aspects to ensure a more comprehensive view of influencing factors in the elite soccer league and soccer games in general.

## Conclusion

The study’s main findings demonstrate that the average actual playing time and average density of EPL games were significantly higher than in the CSL games, showing moderate differences and minor differences, respectively. The average time consumed by general fouls, minor player injuries, and off-field interferences in the CSL games was significantly higher than in the EPL games, while the average time consumed by out-of-bounds was significantly lower than in the EPL. Thus, more anthropogenic factors affected the actual playing time in the CSL than in the EPL, while non-anthropogenic factors affected the two leagues almost similarly. These results provide a reference for coaches to design effective training and develop strategies before a game to enhance players’ ability under high intensity conditions. Furthermore, they can also guide referees to make proper plans for the coming game; thus, securing a more favorable outcome.

## Data Availability Statement

The raw data supporting the conclusions of this article will be made available by the authors, without undue reservation.

## Ethics Statement

Ethical review and approval was not required for the study on human participants in accordance with the local legislation and institutional requirements. Written informed consent from the (patients/participants or patients/participants legal guardian/next of kin) was not required to participate in this study in accordance with the national legislation and the institutional requirements.

## Author Contributions

YZ conceptualized the study, wrote the original draft preparation, and contributed to the methodology and data collection. TL reviewed and edited the manuscript and helped to improve this work. All authors contributed to the article and approved the submitted version.

## Funding

This study was supported by the National Social Science Fund of China (18CTY011).

## Conflict of Interest

The authors declare that the research was conducted in the absence of any commercial or financial relationships that could be construed as a potential conflict of interest.

## Publisher’s Note

All claims expressed in this article are solely those of the authors and do not necessarily represent those of their affiliated organizations, or those of the publisher, the editors and the reviewers. Any product that may be evaluated in this article, or claim that may be made by its manufacturer, is not guaranteed or endorsed by the publisher.

## Supplementary Material

The Supplementary Material for this article can be found online at: https://www.frontiersin.org/articles/10.3389/fpsyg.2022.907336/full#supplementary-material
