# Supplementary material for: Factors That Influence Actual Playing Time: Evidence From the Chinese Super League and English Premier League
Source: Front Psychol. 2022 Jul 4;13:907336. doi: 10.3389/fpsyg.2022.907336 (PMC9289553; doi:10.3389/fpsyg.2022.907336)
Supplement: Supplementary file 2 [file Data_Sheet_1.pdf]

```
RELIABILITY
  /VARIABLES=judge1 judge2
  /SCALE('ALL VARIABLES') ALL
  /MODEL=ALPHA
  /ICC=MODEL(MIXED) TYPE(CONSISTENCY) CIN=95 TESTVAL=0.
```

Reliability

Notes

Output Created		21-MAY-2022 16:12:08
Comments		
Input	Active Dataset	0
	Filter	<none>
	Weight	<none>
	Split File	<none>
	N of Rows in Working Data File	10
	Matrix Input	
Missing Value Handling	Definition of Missing	User-defined missing values are treated as missing.
	Cases Used	Statistics are based on all cases with valid data for all variables in the procedure.
Syntax		RELIABILITY /VARIABLES=judge1 judge2 /SCALE('ALL VARIABLES') ALL /MODEL=ALPHA /ICC=MODEL(MIXED) TYPE(CONSISTENCY) CIN=95 TESTVAL=0.
Resources	Processor Time	00:00:00.00
	Elapsed Time	00:00:00.00

Scale: ALL VARIABLES

Case Processing Summary

		N	%
Cases	Valid	10	100.0
	Excluded[a]	0	.0
	Total	10	100.0

a. Listwise deletion based on all variables in the procedure.

Reliability Statistics

Cronbach's Alpha	N of Items
.995	2

Intraclass Correlation Coefficient

	Intraclass Correlation[b]	95% Confidence Interval		F Test with True Value 0			
		Lower Bound	Upper Bound	Value	df1	df2	Sig
Single Measures	.990[a]	.960	.997	195.707	9	9	.000
Average Measures	.995[c]	.979	.999	195.707	9	9	.000

Two-way mixed effects model where people effects are random and measures effects are fixed.

a. The estimator is the same, whether the interaction effect is present or not.

b. Type C intraclass correlation coefficients using a consistency definition. The between-measure variance is excluded from the denominator variance.

c. This estimate is computed assuming the interaction effect is absent, because it is not estimable otherwise.

```
RELIABILITY
 /VARIABLES=judge1 judge2
 /SCALE('ALL VARIABLES') ALL
 /MODEL=ALPHA
 /ICC=MODEL(MIXED) TYPE(CONSISTENCY) CIN=95 TESTVAL=0.
```

Reliability

Notes

Output Created		21-MAY-2022 16:12:22
Comments		
Input	Active Dataset	0
	Filter	<none>
	Weight	<none>
	Split File	<none>
	N of Rows in Working Data File	10
	Matrix Input	
Missing Value Handling	Definition of Missing	User-defined missing values are treated as missing.
	Cases Used	Statistics are based on all cases with valid data for all variables in the procedure.
Syntax		RELIABILITY /VARIABLES=judge1 judge2 /SCALE('ALL VARIABLES') ALL /MODEL=ALPHA /ICC=MODEL(MIXED) TYPE(CONSISTENCY) CIN=95 TESTVAL=0.
Resources	Processor Time	00:00:00.00
	Elapsed Time	00:00:00.00

Scale: ALL VARIABLES

Case Processing Summary

		N	%
Cases	Valid	10	100.0
	Excluded[a]	0	.0
	Total	10	100.0

a. Listwise deletion based on all variables in the procedure.

Reliability Statistics

Cronbach's Alpha	N of Items
.990	2

Intraclass Correlation Coefficient

	Intraclass Correlation[b]	95% Confidence Interval		F Test with True Value 0			
		Lower Bound	Upper Bound	Value	df1	df2	Sig
Single Measures	.981[a]	.924	.995	102.006	9	9	.000
Average Measures	.990[c]	.961	.998	102.006	9	9	.000

Two-way mixed effects model where people effects are random and measures effects are fixed.

a. The estimator is the same, whether the interaction effect is present or not.

b. Type C intraclass correlation coefficients using a consistency definition. The between-measure variance is excluded from the denominator variance.

c. This estimate is computed assuming the interaction effect is absent, because it is not estimable otherwise.

```
RELIABILITY
 /VARIABLES=judge1 judge2
 /SCALE('ALL VARIABLES') ALL
 /MODEL=ALPHA
 /ICC=MODEL(MIXED) TYPE(CONSISTENCY) CIN=95 TESTVAL=0.
```

Reliability

Notes

Output Created		21-MAY-2022 16:12:35
Comments		
Input	Active Dataset	0
	Filter	<none>
	Weight	<none>
	Split File	<none>
	N of Rows in Working Data File	10
	Matrix Input	
Missing Value Handling	Definition of Missing	User-defined missing values are treated as missing.
	Cases Used	Statistics are based on all cases with valid data for all variables in the procedure.
Syntax		RELIABILITY /VARIABLES=judge1 judge2 /SCALE('ALL VARIABLES') ALL /MODEL=ALPHA /ICC=MODEL(MIXED) TYPE(CONSISTENCY) CIN=95 TESTVAL=0.
Resources	Processor Time	00:00:00.00
	Elapsed Time	00:00:00.00

Scale: ALL VARIABLES

Case Processing Summary

		N	%
Cases	Valid	10	100.0
	Excluded[a]	0	.0
	Total	10	100.0

a. Listwise deletion based on all variables in the procedure.

Reliability Statistics

Cronbach's Alpha	N of Items
.968	2

Intraclass Correlation Coefficient

	Intraclass Correlation[b]	95% Confidence Interval		F Test with True Value 0			
		Lower Bound	Upper Bound	Value	df1	df2	Sig
Single Measures	.939[a]	.775	.984	31.717	9	9	.000
Average Measures	.968[c]	.873	.992	31.717	9	9	.000

Two-way mixed effects model where people effects are random and measures effects are fixed.

a. The estimator is the same, whether the interaction effect is present or not.

b. Type C intraclass correlation coefficients using a consistency definition. The between-measure variance is excluded from the denominator variance.

c. This estimate is computed assuming the interaction effect is absent, because it is not estimable otherwise.

```
RELIABILITY
 /VARIABLES=judge1 judge2
 /SCALE('ALL VARIABLES') ALL
 /MODEL=ALPHA
 /ICC=MODEL(MIXED) TYPE(CONSISTENCY) CIN=95 TESTVAL=0.
```

Reliability

Notes

Output Created		21-MAY-2022 16:12:48
Comments		
Input	Active Dataset	0
	Filter	<none>
	Weight	<none>
	Split File	<none>
	N of Rows in Working Data File	10
	Matrix Input	
Missing Value Handling	Definition of Missing	User-defined missing values are treated as missing.
	Cases Used	Statistics are based on all cases with valid data for all variables in the procedure.
Syntax		RELIABILITY /VARIABLES=judge1 judge2 /SCALE('ALL VARIABLES') ALL /MODEL=ALPHA /ICC=MODEL(MIXED) TYPE(CONSISTENCY) CIN=95 TESTVAL=0.
Resources	Processor Time	00:00:00.00
	Elapsed Time	00:00:00.00

Scale: ALL VARIABLES

Case Processing Summary

		N	%
Cases	Valid	10	100.0
	Excluded[a]	0	.0
	Total	10	100.0

a. Listwise deletion based on all variables in the procedure.

Reliability Statistics

Cronbach's Alpha	N of Items
.977	2

Intraclass Correlation Coefficient

	Intraclass Correlation[b]	95% Confidence Interval		F Test with True Value 0			
		Lower Bound	Upper Bound	Value	df1	df2	Sig
Single Measures	.955[a]	.832	.989	43.918	9	9	.000
Average Measures	.977[c]	.908	.994	43.918	9	9	.000

Two-way mixed effects model where people effects are random and measures effects are fixed.

a. The estimator is the same, whether the interaction effect is present or not.

b. Type C intraclass correlation coefficients using a consistency definition. The between-measure variance is excluded from the denominator variance.

c. This estimate is computed assuming the interaction effect is absent, because it is not estimable otherwise.

```
RELIABILITY
 /VARIABLES=judge1 judge2
 /SCALE('ALL VARIABLES') ALL
 /MODEL=ALPHA
 /ICC=MODEL(MIXED) TYPE(CONSISTENCY) CIN=95 TESTVAL=0.
```

Reliability

Notes

Output Created		21-MAY-2022 16:12:59
Comments		
Input	Active Dataset	0
	Filter	<none>
	Weight	<none>
	Split File	<none>
	N of Rows in Working Data File	10
	Matrix Input	
Missing Value Handling	Definition of Missing	User-defined missing values are treated as missing.
	Cases Used	Statistics are based on all cases with valid data for all variables in the procedure.
Syntax		RELIABILITY /VARIABLES=judge1 judge2 /SCALE('ALL VARIABLES') ALL /MODEL=ALPHA /ICC=MODEL(MIXED) TYPE(CONSISTENCY) CIN=95 TESTVAL=0.
Resources	Processor Time	00:00:00.00
	Elapsed Time	00:00:00.00

Scale: ALL VARIABLES

Case Processing Summary

		N	%
Cases	Valid	10	100.0
	Excluded[a]	0	.0
	Total	10	100.0

a. Listwise deletion based on all variables in the procedure.

Reliability Statistics

Cronbach's Alpha	N of Items
.985	2

Intraclass Correlation Coefficient

	Intraclass Correlation[b]	95% Confidence Interval		F Test with True Value 0			
		Lower Bound	Upper Bound	Value	df1	df2	Sig
Single Measures	.971[a]	.887	.993	66.974	9	9	.000
Average Measures	.985[c]	.940	.996	66.974	9	9	.000

Two-way mixed effects model where people effects are random and measures effects are fixed.

a. The estimator is the same, whether the interaction effect is present or not.

b. Type C intraclass correlation coefficients using a consistency definition. The between-measure variance is excluded from the denominator variance.

c. This estimate is computed assuming the interaction effect is absent, because it is not estimable otherwise.

```
RELIABILITY
 /VARIABLES=judge1 judge2
 /SCALE('ALL VARIABLES') ALL
 /MODEL=ALPHA
 /ICC=MODEL(MIXED) TYPE(CONSISTENCY) CIN=95 TESTVAL=0.
```

Reliability

Notes

Output Created		21-MAY-2022 16:13:11
Comments		
Input	Active Dataset	0
	Filter	<none>
	Weight	<none>
	Split File	<none>
	N of Rows in Working Data File	10
	Matrix Input	
Missing Value Handling	Definition of Missing	User-defined missing values are treated as missing.
	Cases Used	Statistics are based on all cases with valid data for all variables in the procedure.
Syntax		RELIABILITY /VARIABLES=judge1 judge2 /SCALE('ALL VARIABLES') ALL /MODEL=ALPHA /ICC=MODEL(MIXED) TYPE(CONSISTENCY) CIN=95 TESTVAL=0.
Resources	Processor Time	00:00:00.00
	Elapsed Time	00:00:00.00

Scale: ALL VARIABLES

Case Processing Summary

		N	%
Cases	Valid	10	100.0
	Excluded[a]	0	.0
	Total	10	100.0

a. Listwise deletion based on all variables in the procedure.

Reliability Statistics

Cronbach's Alpha	N of Items
.995	2

Intraclass Correlation Coefficient

	Intraclass Correlation[b]	95% Confidence Interval		F Test with True Value 0			
		Lower Bound	Upper Bound	Value	df1	df2	Sig
Single Measures	.989[a]	.957	.997	182.058	9	9	.000
Average Measures	.995[c]	.978	.999	182.058	9	9	.000

Two-way mixed effects model where people effects are random and measures effects are fixed.

a. The estimator is the same, whether the interaction effect is present or not.

b. Type C intraclass correlation coefficients using a consistency definition. The between-measure variance is excluded from the denominator variance.

c. This estimate is computed assuming the interaction effect is absent, because it is not estimable otherwise.

```
RELIABILITY
 /VARIABLES=judge1 judge2
 /SCALE('ALL VARIABLES') ALL
 /MODEL=ALPHA
 /ICC=MODEL(MIXED) TYPE(CONSISTENCY) CIN=95 TESTVAL=0.
```

Reliability

Notes

Output Created		21-MAY-2022 16:13:29
Comments		
Input	Active Dataset	0
	Filter	<none>
	Weight	<none>
	Split File	<none>
	N of Rows in Working Data File	10
	Matrix Input	
Missing Value Handling	Definition of Missing	User-defined missing values are treated as missing.
	Cases Used	Statistics are based on all cases with valid data for all variables in the procedure.
Syntax		RELIABILITY /VARIABLES=judge1 judge2 /SCALE('ALL VARIABLES') ALL /MODEL=ALPHA /ICC=MODEL(MIXED) TYPE(CONSISTENCY) CIN=95 TESTVAL=0.
Resources	Processor Time	00:00:00.00
	Elapsed Time	00:00:00.00

Scale: ALL VARIABLES

Case Processing Summary

		N	%
Cases	Valid	10	100.0
	Excluded[a]	0	.0
	Total	10	100.0

a. Listwise deletion based on all variables in the procedure.

Reliability Statistics

Cronbach's Alpha	N of Items
.999	2

Intraclass Correlation Coefficient

	Intraclass Correlation[b]	95% Confidence Interval		F Test with True Value 0			
		Lower Bound	Upper Bound	Value	df1	df2	Sig
Single Measures	.998[a]	.993	1.000	1079.282	9	9	.000
Average Measures	.999[c]	.996	1.000	1079.282	9	9	.000

Two-way mixed effects model where people effects are random and measures effects are fixed.

a. The estimator is the same, whether the interaction effect is present or not.

b. Type C intraclass correlation coefficients using a consistency definition. The between-measure variance is excluded from the denominator variance.

c. This estimate is computed assuming the interaction effect is absent, because it is not estimable otherwise.

```
RELIABILITY
 /VARIABLES=judge1 judge2
 /SCALE('ALL VARIABLES') ALL
 /MODEL=ALPHA
 /ICC=MODEL(MIXED) TYPE(CONSISTENCY) CIN=95 TESTVAL=0.
```

Reliability

Notes

Output Created		21-MAY-2022 16:13:42
Comments		
Input	Active Dataset	0
	Filter	<none>
	Weight	<none>
	Split File	<none>
	N of Rows in Working Data File	10
	Matrix Input	
Missing Value Handling	Definition of Missing	User-defined missing values are treated as missing.
	Cases Used	Statistics are based on all cases with valid data for all variables in the procedure.
Syntax		RELIABILITY /VARIABLES=judge1 judge2 /SCALE('ALL VARIABLES') ALL /MODEL=ALPHA /ICC=MODEL(MIXED) TYPE(CONSISTENCY) CIN=95 TESTVAL=0.
Resources	Processor Time	00:00:00.00
	Elapsed Time	00:00:00.00

Scale: ALL VARIABLES

Case Processing Summary

		N	%
Cases	Valid	10	100.0
	Excluded[a]	0	.0
	Total	10	100.0

a. Listwise deletion based on all variables in the procedure.

Reliability Statistics

Cronbach's Alpha	N of Items
.993	2

Intraclass Correlation Coefficient

	Intraclass Correlation[b]	95% Confidence Interval		F Test with True Value 0			
		Lower Bound	Upper Bound	Value	df1	df2	Sig
Single Measures	.985[a]	.941	.996	133.419	9	9	.000
Average Measures	.993[c]	.970	.998	133.419	9	9	.000

Two-way mixed effects model where people effects are random and measures effects are fixed.

a. The estimator is the same, whether the interaction effect is present or not.

b. Type C intraclass correlation coefficients using a consistency definition. The between-measure variance is excluded from the denominator variance.

c. This estimate is computed assuming the interaction effect is absent, because it is not estimable otherwise.

```
RELIABILITY
 /VARIABLES=judge1 judge2
 /SCALE('ALL VARIABLES') ALL
 /MODEL=ALPHA
 /ICC=MODEL(MIXED) TYPE(CONSISTENCY) CIN=95 TESTVAL=0.
```

Reliability

Notes

Output Created		21-MAY-2022 16:13:56
Comments		
Input	Active Dataset	0
	Filter	<none>
	Weight	<none>
	Split File	<none>
	N of Rows in Working Data File	10
	Matrix Input	
Missing Value Handling	Definition of Missing	User-defined missing values are treated as missing.
	Cases Used	Statistics are based on all cases with valid data for all variables in the procedure.
Syntax		RELIABILITY /VARIABLES=judge1 judge2 /SCALE('ALL VARIABLES') ALL /MODEL=ALPHA /ICC=MODEL(MIXED) TYPE(CONSISTENCY) CIN=95 TESTVAL=0.
Resources	Processor Time	00:00:00.00
	Elapsed Time	00:00:00.00

Scale: ALL VARIABLES

Case Processing Summary

		N	%
Cases	Valid	10	100.0
	Excluded[a]	0	.0
	Total	10	100.0

a. Listwise deletion based on all variables in the procedure.

Reliability Statistics

Cronbach's Alpha	N of Items
.999	2

Intraclass Correlation Coefficient

	Intraclass Correlation[b]	95% Confidence Interval		F Test with True Value 0			
		Lower Bound	Upper Bound	Value	df1	df2	Sig
Single Measures	.998[a]	.994	1.000	1284.005	9	9	.000
Average Measures	.999[c]	.997	1.000	1284.005	9	9	.000

Two-way mixed effects model where people effects are random and measures effects are fixed.

a. The estimator is the same, whether the interaction effect is present or not.

b. Type C intraclass correlation coefficients using a consistency definition. The between-measure variance is excluded from the denominator variance.

c. This estimate is computed assuming the interaction effect is absent, because it is not estimable otherwise.

```
RELIABILITY
 /VARIABLES=judge1 judge2
 /SCALE('ALL VARIABLES') ALL
 /MODEL=ALPHA
 /ICC=MODEL(MIXED) TYPE(CONSISTENCY) CIN=95 TESTVAL=0.
```

Reliability

Notes

Output Created		21-MAY-2022 16:14:08
Comments		
Input	Active Dataset	0
	Filter	<none>
	Weight	<none>
	Split File	<none>
	N of Rows in Working Data File	10
	Matrix Input	
Missing Value Handling	Definition of Missing	User-defined missing values are treated as missing.
	Cases Used	Statistics are based on all cases with valid data for all variables in the procedure.
Syntax		RELIABILITY /VARIABLES=judge1 judge2 /SCALE('ALL VARIABLES') ALL /MODEL=ALPHA /ICC=MODEL(MIXED) TYPE(CONSISTENCY) CIN=95 TESTVAL=0.
Resources	Processor Time	00:00:00.02
	Elapsed Time	00:00:00.00

Scale: ALL VARIABLES

Case Processing Summary

		N	%
Cases	Valid	10	100.0
	Excluded[a]	0	.0
	Total	10	100.0

a. Listwise deletion based on all variables in the procedure.

Reliability Statistics

Cronbach's Alpha	N of Items
.993	2

Intraclass Correlation Coefficient

	Intraclass Correlation[b]	95% Confidence Interval		F Test with True Value 0			
		Lower Bound	Upper Bound	Value	df1	df2	Sig
Single Measures	.986[a]	.945	.997	142.516	9	9	.000
Average Measures	.993[c]	.972	.998	142.516	9	9	.000

Two-way mixed effects model where people effects are random and measures effects are fixed.

a. The estimator is the same, whether the interaction effect is present or not.

b. Type C intraclass correlation coefficients using a consistency definition. The between-measure variance is excluded from the denominator variance.

c. This estimate is computed assuming the interaction effect is absent, because it is not estimable otherwise.

```
RELIABILITY
 /VARIABLES=judge1 judge2
 /SCALE('ALL VARIABLES') ALL
 /MODEL=ALPHA
 /ICC=MODEL(MIXED) TYPE(CONSISTENCY) CIN=95 TESTVAL=0.
```

Reliability

Notes

Output Created		21-MAY-2022 16:14:20
Comments		
Input	Active Dataset	0
	Filter	<none>
	Weight	<none>
	Split File	<none>
	N of Rows in Working Data File	10
	Matrix Input	
Missing Value Handling	Definition of Missing	User-defined missing values are treated as missing.
	Cases Used	Statistics are based on all cases with valid data for all variables in the procedure.
Syntax		RELIABILITY /VARIABLES=judge1 judge2 /SCALE('ALL VARIABLES') ALL /MODEL=ALPHA /ICC=MODEL(MIXED) TYPE(CONSISTENCY) CIN=95 TESTVAL=0.
Resources	Processor Time	00:00:00.00
	Elapsed Time	00:00:00.00

Scale: ALL VARIABLES

Case Processing Summary

		N	%
Cases	Valid	10	100.0
	Excluded[a]	0	.0
	Total	10	100.0

a. Listwise deletion based on all variables in the procedure.

Reliability Statistics

Cronbach's Alpha	N of Items
.996	2

Intraclass Correlation Coefficient

	Intraclass Correlation[b]	95% Confidence Interval		F Test with True Value 0			
		Lower Bound	Upper Bound	Value	df1	df2	Sig
Single Measures	.993[a]	.972	.998	280.721	9	9	.000
Average Measures	.996[c]	.986	.999	280.721	9	9	.000

Two-way mixed effects model where people effects are random and measures effects are fixed.

a. The estimator is the same, whether the interaction effect is present or not.

b. Type C intraclass correlation coefficients using a consistency definition. The between-measure variance is excluded from the denominator variance.

c. This estimate is computed assuming the interaction effect is absent, because it is not estimable otherwise.

```
RELIABILITY
 /VARIABLES=judge1 judge2
 /SCALE('ALL VARIABLES') ALL
 /MODEL=ALPHA
 /ICC=MODEL(MIXED) TYPE(CONSISTENCY) CIN=95 TESTVAL=0.
```

Reliability

Notes

Output Created		21-MAY-2022 16:14:36
Comments		
Input	Active Dataset	0
	Filter	<none>
	Weight	<none>
	Split File	<none>
	N of Rows in Working Data File	10
	Matrix Input	
Missing Value Handling	Definition of Missing	User-defined missing values are treated as missing.
	Cases Used	Statistics are based on all cases with valid data for all variables in the procedure.
Syntax		RELIABILITY<br>/VARIABLES=judge1 judge2<br>/SCALE('ALL VARIABLES') ALL<br>/MODEL=ALPHA<br>/ICC=MODEL(MIXED) TYPE(CONSISTENCY) CIN=95 TESTVAL=0.
Resources	Processor Time	00:00:00.00
	Elapsed Time	00:00:00.00

Scale: ALL VARIABLES

Case Processing Summary

		N	%
Cases	Valid	10	100.0
	Excluded[a]	0	.0
	Total	10	100.0

a. Listwise deletion based on all variables in the procedure.

Reliability Statistics

Cronbach's Alpha	N of Items
.993	2

Intraclass Correlation Coefficient

	Intraclass Correlation[b]	95% Confidence Interval		F Test with True Value 0			
		Lower Bound	Upper Bound	Value	df1	df2	Sig
Single Measures	.986[a]	.944	.996	140.353	9	9	.000
Average Measures	.993[c]	.971	.998	140.353	9	9	.000

Two-way mixed effects model where people effects are random and measures effects are fixed.

a. The estimator is the same, whether the interaction effect is present or not.

b. Type C intraclass correlation coefficients using a consistency definition. The between-measure variance is excluded from the denominator variance.

c. This estimate is computed assuming the interaction effect is absent, because it is not estimable otherwise.

```
RELIABILITY
 /VARIABLES=judge1 judge2
 /SCALE('ALL VARIABLES') ALL
 /MODEL=ALPHA
 /ICC=MODEL(MIXED) TYPE(CONSISTENCY) CIN=95 TESTVAL=0.
```

Reliability

Notes

Output Created		21-MAY-2022 16:14:48
Comments		
Input	Active Dataset	0
	Filter	<none>
	Weight	<none>
	Split File	<none>
	N of Rows in Working Data File	10
	Matrix Input	
Missing Value Handling	Definition of Missing	User-defined missing values are treated as missing.
	Cases Used	Statistics are based on all cases with valid data for all variables in the procedure.
Syntax		RELIABILITY /VARIABLES=judge1 judge2 /SCALE('ALL VARIABLES') ALL /MODEL=ALPHA /ICC=MODEL(MIXED) TYPE(CONSISTENCY) CIN=95 TESTVAL=0.
Resources	Processor Time	00:00:00.00
	Elapsed Time	00:00:00.00

Scale: ALL VARIABLES

Case Processing Summary

		N	%
Cases	Valid	10	100.0
	Excluded[a]	0	.0
	Total	10	100.0

a. Listwise deletion based on all variables in the procedure.

Reliability Statistics

Cronbach's Alpha	N of Items
1.000	2

Intraclass Correlation Coefficient

	Intraclass Correlation[b]	95% Confidence Interval		F Test with True Value 0			
		Lower Bound	Upper Bound	Value	df1	df2	Sig
Single Measures	1.000[a]	.999	1.000	5556.962	9	9	.000
Average Measures	1.000[c]	.999	1.000	5556.962	9	9	.000

Two-way mixed effects model where people effects are random and measures effects are fixed.

a. The estimator is the same, whether the interaction effect is present or not.

b. Type C intraclass correlation coefficients using a consistency definition. The between-measure variance is excluded from the denominator variance.

c. This estimate is computed assuming the interaction effect is absent, because it is not estimable otherwise.

```
RELIABILITY
 /VARIABLES=judge1 judge2
 /SCALE('ALL VARIABLES') ALL
 /MODEL=ALPHA
 /ICC=MODEL(MIXED) TYPE(CONSISTENCY) CIN=95 TESTVAL=0.
```

Reliability

Notes

Output Created		21-MAY-2022 16:15:01
Comments		
Input	Active Dataset	0
	Filter	<none>
	Weight	<none>
	Split File	<none>
	N of Rows in Working Data File	10
	Matrix Input	
Missing Value Handling	Definition of Missing	User-defined missing values are treated as missing.
	Cases Used	Statistics are based on all cases with valid data for all variables in the procedure.
Syntax		RELIABILITY /VARIABLES=judge1 judge2 /SCALE('ALL VARIABLES') ALL /MODEL=ALPHA /ICC=MODEL(MIXED) TYPE(CONSISTENCY) CIN=95 TESTVAL=0.
Resources	Processor Time	00:00:00.00
	Elapsed Time	00:00:00.00

Scale: ALL VARIABLES

Case Processing Summary

		N	%
Cases	Valid	10	100.0
	Excluded[a]	0	.0
	Total	10	100.0

a. Listwise deletion based on all variables in the procedure.

Reliability Statistics

Cronbach's Alpha	N of Items
.991	2

Intraclass Correlation Coefficient

	Intraclass Correlation[b]	95% Confidence Interval		F Test with True Value 0			
		Lower Bound	Upper Bound	Value	df1	df2	Sig
Single Measures	.983[a]	.934	.996	117.326	9	9	.000
Average Measures	.991[c]	.966	.998	117.326	9	9	.000

Two-way mixed effects model where people effects are random and measures effects are fixed.

a. The estimator is the same, whether the interaction effect is present or not.

b. Type C intraclass correlation coefficients using a consistency definition. The between-measure variance is excluded from the denominator variance.

c. This estimate is computed assuming the interaction effect is absent, because it is not estimable otherwise.

```
RELIABILITY
 /VARIABLES=judge1 judge2
 /SCALE('ALL VARIABLES') ALL
 /MODEL=ALPHA
 /ICC=MODEL(MIXED) TYPE(CONSISTENCY) CIN=95 TESTVAL=0.
```

Reliability

Notes

Output Created		21-MAY-2022 16:15:13
Comments		
Input	Active Dataset	0
	Filter	<none>
	Weight	<none>
	Split File	<none>
	N of Rows in Working Data File	10
	Matrix Input	
Missing Value Handling	Definition of Missing	User-defined missing values are treated as missing.
	Cases Used	Statistics are based on all cases with valid data for all variables in the procedure.
Syntax		RELIABILITY /VARIABLES=judge1 judge2 /SCALE('ALL VARIABLES') ALL /MODEL=ALPHA /ICC=MODEL(MIXED) TYPE(CONSISTENCY) CIN=95 TESTVAL=0.
Resources	Processor Time	00:00:00.00
	Elapsed Time	00:00:00.00

Scale: ALL VARIABLES

Case Processing Summary

		N	%
Cases	Valid	10	100.0
	Excluded[a]	0	.0
	Total	10	100.0

a. Listwise deletion based on all variables in the procedure.

Reliability Statistics

Cronbach's Alpha	N of Items
1.000	2

Intraclass Correlation Coefficient

	Intraclass Correlation[b]	95% Confidence Interval		F Test with True Value 0			
		Lower Bound	Upper Bound	Value	df1	df2	Sig
Single Measures	1.000[a]	1.000	1.000	.	9	.	.
Average Measures	1.000[c]	1.000	1.000	.	9	.	.

Two-way mixed effects model where people effects are random and measures effects are fixed.

a. The estimator is the same, whether the interaction effect is present or not.

b. Type C intraclass correlation coefficients using a consistency definition. The between-measure variance is excluded from the denominator variance.

c. This estimate is computed assuming the interaction effect is absent, because it is not estimable otherwise.

```
RELIABILITY
 /VARIABLES=judge1 judge2
 /SCALE('ALL VARIABLES') ALL
 /MODEL=ALPHA
 /ICC=MODEL(MIXED) TYPE(CONSISTENCY) CIN=95 TESTVAL=0.
```

Reliability

Notes

Output Created		21-MAY-2022 16:15:27
Comments		
Input	Active Dataset	0
	Filter	<none>
	Weight	<none>
	Split File	<none>
	N of Rows in Working Data File	10
	Matrix Input	
Missing Value Handling	Definition of Missing	User-defined missing values are treated as missing.
	Cases Used	Statistics are based on all cases with valid data for all variables in the procedure.
Syntax		RELIABILITY<br>/VARIABLES=judge1 judge2<br>/SCALE('ALL VARIABLES') ALL<br>/MODEL=ALPHA<br>/ICC=MODEL(MIXED) TYPE(CONSISTENCY) CIN=95 TESTVAL=0.
Resources	Processor Time	00:00:00.00
	Elapsed Time	00:00:00.00

Scale: ALL VARIABLES

Case Processing Summary

		N	%
Cases	Valid	10	100.0
	Excluded[a]	0	.0
	Total	10	100.0

a. Listwise deletion based on all variables in the procedure.

Reliability Statistics

Cronbach's Alpha	N of Items
.999	2

Intraclass Correlation Coefficient

	Intraclass Correlation[b]	95% Confidence Interval		F Test with True Value 0			
		Lower Bound	Upper Bound	Value	df1	df2	Sig
Single Measures	.999[a]	.995	1.000	1747.667	9	9	.000
Average Measures	.999[c]	.998	1.000	1747.667	9	9	.000

Two-way mixed effects model where people effects are random and measures effects are fixed.

a. The estimator is the same, whether the interaction effect is present or not.

b. Type C intraclass correlation coefficients using a consistency definition. The between-measure variance is excluded from the denominator variance.

c. This estimate is computed assuming the interaction effect is absent, because it is not estimable otherwise.

```
RELIABILITY
 /VARIABLES=judge1 judge2
 /SCALE('ALL VARIABLES') ALL
 /MODEL=ALPHA
 /ICC=MODEL(MIXED) TYPE(CONSISTENCY) CIN=95 TESTVAL=0.
```

Reliability

Notes

Output Created		21-MAY-2022 16:15:38
Comments		
Input	Active Dataset	0
	Filter	<none>
	Weight	<none>
	Split File	<none>
	N of Rows in Working Data File	10
	Matrix Input	
Missing Value Handling	Definition of Missing	User-defined missing values are treated as missing.
	Cases Used	Statistics are based on all cases with valid data for all variables in the procedure.
Syntax		RELIABILITY /VARIABLES=judge1 judge2 /SCALE('ALL VARIABLES') ALL /MODEL=ALPHA /ICC=MODEL(MIXED) TYPE(CONSISTENCY) CIN=95 TESTVAL=0.
Resources	Processor Time	00:00:00.00
	Elapsed Time	00:00:00.00

Scale: ALL VARIABLES

Case Processing Summary

		N	%
Cases	Valid	10	100.0
	Excluded[a]	0	.0
	Total	10	100.0

a. Listwise deletion based on all variables in the procedure.

Reliability Statistics

Cronbach's Alpha	N of Items
.999	2

Intraclass Correlation Coefficient

	Intraclass Correlation[b]	95% Confidence Interval		F Test with True Value 0			
		Lower Bound	Upper Bound	Value	df1	df2	Sig
Single Measures	.999[a]	.995	1.000	1714.103	9	9	.000
Average Measures	.999[c]	.998	1.000	1714.103	9	9	.000

Two-way mixed effects model where people effects are random and measures effects are fixed.

a. The estimator is the same, whether the interaction effect is present or not.

b. Type C intraclass correlation coefficients using a consistency definition. The between-measure variance is excluded from the denominator variance.

c. This estimate is computed assuming the interaction effect is absent, because it is not estimable otherwise.
